# Reducing Venereal Disease in Belgium

**Published:** 1924-06

**Authors:** 


### REDUCING VENEREAL DISEASE IN BELGIUM.

Dr. R. Bernard, who is in charge of a venereal disease dispensary in Brussels, has recently published a remarkable paper on the decline of syphilis in Belgium, following the intensive campaign conducted in that country during the last few years against venereal disease. Dr. Bernard's figures deal with the work of his dispensary during the past five years. In 1920 there were 108 cases of syphilis observed in the primary stage. This figure fell to 38 in 1921, and to 12 in 1922. In the same period there was a marked decline also in the number of cases of secondary syphilis, and the ratio of primary cases to all the cases of syphilis fell from 35.64 per cent. in 1920, and to 8.42 per cent. in 1923. One of the administrative measures which have helped to bring down the incidence of syphilis with a run in Belgium is the supply by the Government of the salvarsan group of drugs free of charge to every medical practitioner, who is thus enabled to give the best available specific treatment to the most indigent of his patients.

